# Persistence of vaccine-induced antibodies to A/H5N1 at 30 months and 36 months after vaccination in Vietnam

**DOI:** 10.4178/epih.e2021076

**Published:** 2021-10-06

**Authors:** Chien Vien Chinh, Viet Phu Quoc, Loc Huynh Tan, Duoc Nguyen Van, Thai Pham Quang, Be Le Van

**Affiliations:** 1 Institute of Vaccines and Medical Biologicals (IVAC), Nha Trang, Vietnam; 2 Tay Nguyen Institute of Hygiene and Epidemiology, Buon Me Thuot, Vietnam; 3 Khanh Hoa Medical Service, Nha Trang, Vietnam; 4 Ninh Hoa District Medical Center, Khanh Hoa, Vietnam; 5 National Institute of Hygiene and Epidemiology, Hanoi, Vietnam; 6 School of Preventive Medicine and Public Health, Hanoi Medical University, Hanoi, Vietnam

**Keywords:** Antibody persistence, Influenza A/H5N1, Vietnam, Influenza vaccine

## Abstract

**OBJECTIVES:**

An A/H5N1 vaccine (IVACFLU-A/H5N1) was accepted for use in Vietnam; however, antibody persistence after vaccination has not been well characterized. We examined post-vaccination antibody persistence and related risk factors in individuals enrolled in the phase II IVACFLU-A/H5N1 vaccine trial in Ninh Hoa, Vietnam, who received a 15-μg dose (2 injections 21 days apart).

**METHODS:**

We used a longitudinal study design to follow 86 participants, without a control group. The participants tested as anti-A/H5N1 immunoglobulin G seronegative at baseline and received both doses of the vaccine. Blood was drawn at 30 months and 36 months after the complete vaccination to assess antibody status. Antibody persistence status was compared by demographic characteristics and exposure risk factors using univariate logistic regression.

**RESULTS:**

In total, 84.9% and 52.3% of the population showed persistence of at least 1/10 of the A/H5N1 antibodies at 30 months and 36 months after IVACFLU-A/H5N1 vaccination, respectively. The odds of antibody persistence were higher in older people, but lower in people who had experienced flu-like symptoms in the past 18 months or between 2 visits. We recorded no differences between A/H5N1 antibody persistence and exposure risk factors, including having a poultry farm, coming into contact with poultry, and slaughtering and processing poultry.

**CONCLUSIONS:**

This study demonstrated noteworthy antibody persistence, indicated by the seroconversion rate and geometric mean titer at 30 months and 36 months after the IVACFLU-A/H5N1 vaccine. Further studies should investigate older people and those who experienced flu-like symptoms to determine a suitable time for a booster shot.

## INTRODUCTION

H5N1 is a type of influenza virus that causes highly infectious, severe respiratory disease in birds, known as avian influenza. The H5N1 bird flu incident in Hong Kong in 1997 was the first known instance of a purely avian virus causing severe human disease and death. Widespread re-emergence of avian influenza occurred in 2003 in Asia and later in Africa, Europe, and the Middle East. Influenza A virus subtype H5N1 (A/H5N1) causes illness in humans and many other animal species. Vietnam is 1 of 6 countries that were identified as A/H5N1 hotspots at that time. From 2003 to 2009, the World Health Organization (WHO) recorded 861 humans infected with H5N1 and 455 deaths, of which 50.4% occurred in Vietnam [1].

Vaccines remain one of our best defense weapons against the influenza virus. In 2006, the WHO and the wider international public health community launched the Global Action Plan for Influenza Vaccines to help ramp up the production capacity of vaccination in 14 countries, including Vietnam [2]. In 2012, the Institute of Vaccines and Medical Biologicals (IVAC) successfully produced an influenza vaccine named IVACFLU-A/H5N1, which constitutes a whole-virion vaccine produced by technology involving chicken embryos, with utilization of egg micronutrients, formalin inactivation, adsorption of aluminum hydroxide, and no preservatives. Evidence from 1,005 Vietnamese people who voluntarily received injections showed that this vaccine produced a high seroconversion rate (75.8%), equivalent to a high level of antibodies after vaccination [3].

Antibody persistence, which is widely defined as the maintenance of seropositivity, is vital for sustained protection [4]. The persistence of vaccine antibodies decreases over time and depends on several factors, such as age, sex, and the vaccine’s components (e.g., adjuvants) [5]. To our knowledge, there is limited evidence on the relationships among factors related to antibody persistence rate over time. IVACFLU-A/H5N1 was documented as effective in Vietnam; however, antibody persistence after vaccination has not been well characterized due to the recent approval of the vaccine. A study in the United States showed that the antibody rate was 76.7% at 6 months after vaccination with an ASO_3A_-adjuvanted H5N1 vaccine [6]; this figure decreased to 30.9% and 8.3% after 2 years for vaccine formulations with and without adjuvants, respectively [5]. Other evidence suggested that antibody rates persisted for more than 2 years after H5N1 vaccination [7].

We examined the persistence of antibodies after vaccination and related risk factors in individuals enrolled in the phase II IVACFLU-A/H5N1 vaccine trial in Ninh Hoa, Vietnam, who received a 15-μg dose (2 injections 21 days apart). Our study is one of the first to explore long-term antibody persistence and its associated characteristics in IVACFLU-A/H5N1 vaccinated individuals.

## MATERIALS AND METHODS

### Participant selection and enrollment

**Figure 1 f1-epih-43-e2021076:**
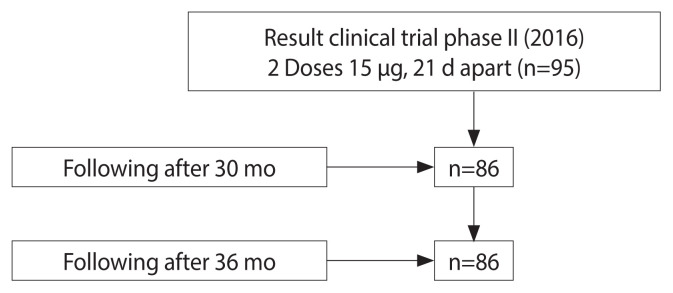
Participant flow.

We conducted a longitudinal study without a control group in the Ninh Hoa commune, Khanh Hoa Province, Vietnam. The phase II clinical trial of a recombinant IVACFLU-A/H5N1 vaccine in 2016 included 194 participants from the Ninh Hoa commune, Khanh Hoa Province, Vietnam who received an A/H5N1 vaccine (experimental IVACFLU-A/H5N1). Of the participants, 95 received 2 doses of 15 μg and 99 received 2 doses of 30 μg. At the end of phase II, the 15-μg dose was selected for the commercial vaccine. We invited all 95 participants from phase II who received a dose of 15 μg to join this study; 91.6% (87/95) agreed, yielding a final sample of 86 (Figure 1). To be eligible for inclusion in this follow-up study, the participants were required to have tested as anti-A/H5N1 immunoglobulin G (IgG) seronegative at baseline. They subsequently received both doses of the vaccine. Blood was drawn at 30-month and 36-month after the complete vaccination to assess antibody status.

The vaccine is produced from the NIBRG-14 vaccine strain, which is derived from the recombinant A/Vietnam/1194/2004 strain recombinant with A/PR/8/34 influenza, taking the hemagglutinin and neuraminidase genes of A/VN/1194/2004, from which the toxic components were removed to reduce virulence. The genome of influenza A/PR/8/34 came from the National Institute for Biological Standards and Control of the Health Protection Agency, England. [Fig f1-epih-43-e2021076]

In 2018 and 2019, at 30 months after complete vaccination, a 3-mL venous blood draw was taken from each participant, and a designed questionnaire assessing potential A/H5N1 exposures was administered by trained study personnel. After being drawn, the blood was immediately centrifuged, and the resulted serum was separated and frozen at −20°C.

### A/H5N1 virus antibody testing

After completing the follow-up appointments, the serum samples were shipped to the IVAC in Nha Trang, Vietnam, where they were tested for anti-A/H5N1 IgG antibodies using the hemagglutination inhibition (HI) assay. This assay titrated the antibody response to a viral infection, which is currently the only accepted assay of predicting protection against influenza virus [8]. In addition, this assay takes advantage of some viruses’ ability to bind red blood cells. The HI assay was carried out in 96 plates that contained 2-fold dilutions of the sera. Researchers added a known titer of the virus to these plates and incubated them for 30 minutes at 68–77°F (20–25°C). They then added more red blood cells and left them for 30 minutes at the same temperature. The antibodies bind and prevent the virus from hemagglutinating the red blood cells if they are present in the sera sample, cross-reacting with the virus. In this way, we determined the exact titer of the antibodies in the sera [9].

The HI antibody parameters included geometric mean titer (GMT) and seroconversion rate, which is defined as the percentage of subjects achieving an increase in titer from <1:10 before to ≥1:40 after receiving the vaccine, or at least a 4-fold increase in titer from a pre-vaccination titer ≥1:10.

### Statistical analysis

We used Epi Info version 6 for statistical analyses. After completing the anti-A/H5N1 test, we assessed whether each individual showed persistent 1/10 levels of antibodies at 30 months and 36 months after complete vaccination compared to baseline. Antibody persistence status (persistence of a 1/10 level of antibodies at 30 months and 36 months) was compared according to demographic characteristics and exposure risk factors using univariate logistic regression. One month after receipt of the second vaccine dose was used as the time of seroconversion. We calculated the GMT (the average antibody titer) by multiplying all values and taking the 86th root of this number (86 was the number of participants in our study).

### Ethics statement

The Institutional Review Board of the Khanh Hoa Department of Health approved this study with the accreditation number 03/HDDD-SYT on August 30, 2018. All participants provided informed written consent before all follow-up visits. The Ethics Committee of the Khanh Hoa Department of Health approved all procedures for the ongoing follow-up after the vaccine trial.

## RESULTS

In this study, we found that 84.9% and 52.3% of participants showed persistence of at least a 1/10 level of A/H5N1 antibodies at 30 months and 36 months after complete vaccination, respectively. More than half (57.0%) of participants showed seroconversion at 30 months, with levels at least 4 times higher than baseline. Meanwhile, this proportion at 36 months was 30.2%. The GMTs (average antibody titers) at 30 months and 36 months were 4 times and 2 times higher than baseline, respectively (Table 1).

Table 2 presents the demographic characteristics, personal behaviors, medical history, and exposure risk factors of those who showed persistence of at least a 1/10 level of A/H5N1 antibodies at 30 months and 36 months. The proportions of male with persistence of at least a 1/10 level of antibodies at 30 months and 36 months after completing the full IVACFLU-A/H5N1 vaccination were 73.9% and 43.5%, while these figures in female were 88.9% and 55.6%, respectively. In our study, sex was not significantly associated with the persistence of at least a 1/10 level of antibodies at both 30 months and 36 months after vaccination. Older people (41–65 years) had higher odds of persistence of at least a 1/10 level of A/H5N1 antibodies than younger participants (18–40 years; p=0.02) (using participants’ age at the day of vaccination in 2016). In contrast, people who experienced flu-like symptoms in the past 18 months or between 2 visits had lower odds of persistence of at least a 1/10 level of A/H5N1 antibodies than those who did not (p=0.02).

Our study did not find associations between persistence of at least a 1/10 level of antibodies and personal behaviors (alcohol use and smoking) or exposure risk factors (having a poultry farm, directly contacting poultry, and directly slaughtering and processing poultry).

## DISCUSSION

The overall proportion of participants with persistence of at least a 1/10 level of A/H5N1 antibodies was 84.9% and 52.3% at 30 months and 36 months after IVACFLU-A/H5N1 vaccination, respectively. The GMT of participants at 30 months and 36 months post-vaccination was 22.3 and 12.0, respectively. Antibody persistence was greater in older people (odds ratio [OR], 5.65; 95% confidence interval [CI], 1.06 to 39.87; p=0.02). However, the estimate of antibody persistence after vaccination was lower in people who experienced flu-like symptoms in the past 18 months or between 2 visits (OR, 0.09; 95% CI, 0.01 to 0.81; p=0.02).

We found that most participants (84.9%) showed persistence of at least a 1/10 level of A/H5N1 antibodies, and the proportion of those with seroconversion at 30 months after vaccination was 57.0%. At 36 months after vaccination, the former figure decreased to 52.3% and the latter declined to 30.2% Subjects with a titer of ≥1:10 can be considered seropositive. However, opinions on protective titers for the influenza vaccine are quite diverse. Classic research, supported by more recent studies, has established a correlation between the antibody titer evaluated by the HI assay and protection against influenza virus infection, with an HI titer of 1:40 equivalent to 50% protection against infection [10–12]. Lower titers, such as 1:32 or 1:20, have previously been used for HI and neutralizing antibodies to characterize the immune responses to influenza vaccines [13–15]. Meanwhile, other studies have argued that there is insufficient evidence to deduce that an HI titer of 1:40 predicts 50% protection against influenza virus infection [16,17]. Therefore, further research on IVACFLU-A/H5N1 should be conducted to establish a time point for booster vaccination. Nevertheless, it is noteworthy that these results were obtained at 30 months and 36 months post-vaccination without booster shots, indicating a relatively high persistence of antibodies [18].

Our findings on the GMT (the average antibody titer) persisting over time are remarkable compared to previous studies on other H5N1 vaccines. Comparing to a study by Gillard et al. [18] on ASO_3A_-adjuvanted heterologous H5N1 vaccines, the GMT at 36 months post-vaccination was comparable (12.0 vs. 10.5). At the same time, our figure for 30 months was notably higher despite a longer follow-up time (30 vs. 12, 18, 24, and 30 months). In another study on an ASO_3A_-adjuvanted A/H5N1 vaccine, the GMT for 12 months following vaccination was reported to be 11.8, which is considerably lower than our results at both 30 months and 36 months [19]. Aside from the physiological condition of the study subjects, differences in the results of immunological indicators between studies might be attributable to variation in vaccine composition, particularly adjuvants. Adjuvants have been proven to play an important role in enhancing immunogenicity through either physical or chemical associations with antigens [20]. Although evidence has demonstrated the safety and effectiveness of ASO_3_ in boosting the immune response, aluminum-based adjuvants have remained the “gold standard” for adjuvants due to their long-term success [21]. The results observed in this study suggest that future research should compare the performance of ASO_3_ and aluminum-based adjuvants in terms of the persistence of immune responses elicited by the A/H5N1 vaccine.

Statistically significant associations were found in this study between persistence of at least a 1/10 level of A/H5N1 antibodies and older age (OR, 5.65), the experience of flu-like symptoms in the past 18 months or between 2 visits (OR, 0.09). However, notwithstanding previous findings of the persistence of influenza antibodies in the elderly [22,23], there remains no specific evidence or explanation for these findings in the age groups examined in this study (18–40 and 41–65 years old). Meanwhile, the finding of lower antibody persistence in people with flu-like symptoms and the mechanisms underlying this relationship need more investigation.

### Clinical recommendations

The IVACFLU-A/H5N1 vaccine, after 2 injections of 15 μg, showed persistent antibody residues for up to 30 months (84.9%) and after 36 months (52.3%). The IVACFLU-A/H5N1 vaccine does not affect common influenza epidemiology characteristic, except that it may reduce the risk of infection with common influenza within 18 months.

### Limitations and strengths

The research was conducted with a small sample, so there are still many inconclusive points. Therefore, longer monitoring of antibody retention time is required, and further research should include a larger sample to study the epidemiological characteristics associated with antibody persistence after IVACFLU-A/H5N1 vaccination.

In conclusion, this study highlights the antibody persistence (indicated by the seroconversion rate and GMT) at 30 months and 36 months after the IVACFLU-A/H5N1 vaccine. Antibody persistence was significantly more likely in participants aged 41–65, but less likely in people who experienced flu-like symptoms in the past 18 months or between 2 visits. We recorded no differences between A/H5N1 antibody persistence and the exposure of risk factors such as working at poultry farm, poultry contact, poultry slaughtering, or poultry processing.

## Figures and Tables

**Table 1:** Prevalence of persistence of at least a 1/10 amount of A/H5N1 antibodies and seroconversion (≥4 times higher levels compared to baseline) among participants

Time	Persistence of at least a 1/10 amount of antibodies, n (%)	Seroconversion (≥4 times higher than baseline), n (%)	Geometric mean titer^1^	Geometric mean titer ratio^1^
Baseline^2^	11 (12.8)	-	5.6	1.0
21 d after full vaccination (42 d after the first dose)	86 (100)	81 (92.4)	66.8	11.8
30 mo^3^	73 (84.9)	49 (57.0)	22.3	4.0
36 mo^3^	45 (52.3)	26 (30.2)	12.0	2.1

1 Immunological indicators.

2 Before receiving the IVACFLU-A/H5N1 vaccine.

3 At 30 months and 36 months after receiving both IVACFLU-A/H5N1 vaccines – full vaccination.

**Table 2:** Demographic characteristics, personal behaviors, medical history, and exposure risk factors for persistence of at least a 1/10 amount of A/H5N1 antibodies at 30 months and 36 months

Characteristics	Total	30 mo			36 mo		
		
	n (%)	n (%)	OR (95% CI)^1^	p-value	n (%)	OR (95% CI)^1^	p-value
Demographics							
Sex							
Male	23 (26.7)	17 (73.9)	0.35 (0.09, 1.40)	0.09	10 (43.5)	0.62 (0.21, 1.78)	0.32
Female	63 (73.3)	56 (88.9)	1.00 (reference)		35 (55.6)	1.00 (reference)	
Age (yr)							
18–40	39 (45.3)	37 (94.9)	5.65 (1.06, 39.87)	0.02	24 (61.5)	1.98 (0.77, 5.17)	0.12
41–65	47 (54.7)	36 (76.6)	1.00 (reference)		21 (44.7)	1.00 (reference)	

Personal behaviors							
Alcohol use							
Yes	17 (19.8)	14 (82.4)	0.79 (0.17, 4.19)	0.74	10 (58.8)	1.39 (0.42, 4.64)	0.55
No	69 (80.2)	59 (85.5)	1.00 (reference)		35 (50.7)	1.00 (reference)	
Smoking							
Yes	14 (16.3)	11 (78.6)	0.59 (0.12, 3.22)	0.75	6 (42.9)	0.63 (0.17, 2.29)	0.44
No	72 (83.7)	62 (86.1)	1.00 (reference)		39 (54.2)	1.00 (reference)	

Medical history							
Having flu-like symptoms in the past 18 mo or between 2 visits							
Yes	33 (38.4)	29 (87.9)	1.19 (0.32, 4.59)	0.78	1 (11.1)	0.09 (0.01, 0.81)	0.02
No	53 (61.6)	44 (83.0)	1.00 (reference)		44 (57.1)	1.00 (reference)	
Having other diseases in the past 18 mo or between 2 visits							
Yes	7 (8.1)	7 (100)	-	0.53	0 (0.0)	-	0.43
No	79 (91.9)	66 (83.5)	-		45 (53.6)	-	

Exposure risk factors							
Having a poultry farm							
Yes	53 (61.6)	44 (83.0)	0.70 (0.16, 2.72)	0.76	30 (56.6)	1.57 (0.60, 4.12)	0.31
No	33 (38.4)	29 (87.9)	1.00 (reference)		15 (45.5)	1.00 (reference)	
Coming into direct contact with poultry							
Yes	49 (57.0)	41 (83.7)	0.80 (0.20, 3.06)	0.72	28 (57.1)	1.57 (0.61, 4.06)	0.30
No	37 (43.0)	32 (86.5)	1.00 (reference)		17 (45.9)	1.00 (reference)	
Directly slaughtering and processing poultry							
Yes	5 (5.8)	5 (100)	-	0.33	1 (20.0)	0.21 (0.01, 2.15)	0.30
No	81 (94.2)	68 (84.0)	-		44 (54.3)	1.00 (reference)	

OR, odds ratio; CI, confidence interval.

1 Univariate logistic regression models.
